# A Selective Ultrahigh Responding High Temperature Ethanol Sensor Using TiO_2_ Nanoparticles

**DOI:** 10.3390/s140813613

**Published:** 2014-07-28

**Authors:** M. M. Arafat, A. S. M. A. Haseeb, Sheikh A. Akbar

**Affiliations:** 1 Department of Mechanical Engineering, Faculty of Engineering, University of Malaya, 50603 Kuala Lumpur, Malaysia; E-Mail: arafat_mahmood@siswa.um.edu.my; 2 Department of Materials Science and Engineering, The Ohio State University, 2041 College Road, Columbus, OH 43210, USA; E-Mail: akbar.1@osu.edu

**Keywords:** sensor, TiO_2_ nanoparticles, ethanol sensing, catalytic activity

## Abstract

In this research work, the sensitivity of TiO_2_ nanoparticles towards C_2_H_5_OH, H_2_ and CH_4_ gases was investigated. The morphology and phase content of the particles was preserved during sensing tests by prior heat treatment of the samples at temperatures as high as 750 °C and 1000 °C. Field emission scanning electron microscopy (FESEM), transmission electron microscopy (TEM) and X-ray diffraction (XRD) analysis were employed to characterize the size, morphology and phase content of the particles. For sensor fabrication, a film of TiO_2_ was printed on a Au interdigitated alumina substrate. The sensing temperature was varied from 450 °C to 650 °C with varying concentrations of target gases. Results show that the sensor has ultrahigh response towards ethanol (C_2_H_5_OH) compared to hydrogen (H_2_) and methane (CH_4_). The optimum sensing temperature was found to be 600 °C. The response and recovery times of the sensor are 3 min and 15 min, respectively, for 20 ppm C_2_H_5_OH at the optimum operating temperature of 600 °C. It is proposed that the catalytic action of TiO_2_ with C_2_H_5_OH is the reason for the ultrahigh response of the sensor.

## 1. Introduction

Increasing demand for better control of environmental monitoring of emissions from industry and automobiles, improved processing of food and pharmaceuticals, healthcare and weather prediction require high performance gas sensors. Monitoring colorless organic compounds is a growing need in many industries due to possible health and safety concerns [1]. In many applications, ethanol (C_2_H_5_OH) sensors are being used to monitor chemical reactions, biomedical productions, quality control of food and beverages, as well as breath analysis [2,3]. Increased usage of C_2_H_5_OH raises concerns over groundwater pollution [4] and explosion hazards [5]. Thermodynamic analysis shows that C_2_H_5_OH reforms to methane (CH_4_) at moderate temperatures, whereas hydrogen rich gases are formed at high temperatures (427–527 °C) [6–8]. For this reason, the need for selective sensing of C_2_H_5_OH at high temperatures in presence of H_2_ and CH_4_ is attracting the attention of researchers.

Semiconducting metal oxides are being used as gas sensing materials due to their numerous benefits such as high sensitivity, easy fabrication process and low cost [9]. So far, a great variety of metal oxides such as ZnO, SnO_2_, TiO_2_, In_2_O_3_, WO_x_, AgVO_3_, CdO, MoO_3_, CuO, NiO and TeO_2_ have been investigated as sensing materials. Many of these metal oxides exhibit good sensitivity at low temperatures. A review of the literature reveals that ZnO, SnO_2_, In_2_O_3_, WO_x_, AgVO_3_, CdO, MoO_3_, CuO and TeO_2_ have optimum sensitivity at temperatures below 400°C, whereas TiO_2_ is capable of operating at temperatures as high as 600 °C [10]. Moreover, while low temperature gas sensing materials may undergo morphological and phase changes at high sensing temperatures, TiO_2_ is stable at high operating temperatures. Additionally, the catalytic activity of TiO_2_ towards alcohols offers higher electron exchange which is beneficial for C_2_H_5_OH sensing [11,12]. Non-toxicity, easy fabrication and low cost are additional benefits of TiO_2_ in high temperature gas sensing applications.

So far, thick film [13,14], thin film [15] and nanomaterials [16] based on TiO_2_ have been utilized for gas sensing applications. Increasing the surface-to-volume ratio is considered to be an effective way to improve the performance of the gas sensing devices. In particular, reduction of the grain size down to the nanometer level has been suggested as an efficient strategy to enhance the gas-sensing properties of metal oxides [17]. Availability of higher density gas adsorption sites in nano-crystalline materials is the possible reason for the high sensitivity of nanomaterials towards gas sensing [18,19].

A great variety of TiO_2_ nanomaterials have been used for detecting different gases. These include nanowires [20,21], nanotubes [22–25], nanofibers [26–28], nanobelts [29], spherical colloids [30] and nanoparticles [17,31]. However, in the literature TiO_2_ nanostructures with mostly the anatase phase have been discussed for C_2_H_5_OH sensing. It is likely that the anatase phase can transform to rutile during sensing at high temperatures. Phase changes of the material during sensing affect the reproducibility of the results. Stabilization of the phase content at temperatures higher than the sensing temperatures was ignored in most of the studies described in the literature.

In this work, commercial TiO_2_ nanoparticles were used for investigating their sensitivity towards C_2_H_5_OH, H_2_ and CH_4_ gases. An easy processing route was employed for the fabrication of gas sensors containing a porous film of TiO_2_ nanoparticles. Before starting the sensing experiments, the morphology and phase content of TiO_2_ nanoparticles were preserved by heat treating the particles at higher temperatures. Sensitivity, selectivity, optimum temperature, response time and recovery time were then determined and are reported here.

## 2. Experimental Section

Commercial TiO_2_ nanoparticles purchased from Sigma Aldrich (USA) were used in this study (<100 nm (BET)). To stabilize the phase content, a portion of the as-received nanoparticles were heat treated at 750 °C and 1000 °C for 3 h and 1 h, respectively. Heat treatment was conducted in Ar and ambient gas environments. The morphology and size of the particles before and after the heat treatment were measured using a high resolution field emission scanning electron microscope (FESEM: Zeiss Ultra-60, Atlanta, GA, USA) and a transmission electron microscope (HRTEM: FEI Tecnai F-20, Hillsboro, OR, USA). The phase content in the particles were characterized by an X-ray diffractometer (XRD: PANalytical Empyrean, Netherlands) using CuK_α_ radiation (0.1540598 nm) at 40 KV and 40 mA.

An ink was formulated to disperse the particles on the substrate. For this, α-terpineol (Sigma Aldrich) and diethylene glycol dibutyl ether (DGDE: Sigma Aldrich) were mixed at a ratio of 1:1 at room temperature. Then 8 wt% of ethyl cellulose (Sigma Aldrich) was added to the solution and heated at 200 °C for 2 hours to dissolve the ethyl cellulose. Then the ink was loaded with 10 wt% TiO_2_ nanoparticles. The composite ink was ultrasonicated for 1 hour to disperse the particles. About 3 μL of the composite ink was taken by a micropipette and dropped on to an Au interdigitated alumina substrate having a dimension of 5 mm × 5 mm (purchased from Case Western Reserve University, Cleveland, OH, USA). After that the sensors were heat treated at 750 °C for 1 hour in Ar atmosphere at which the phase contents were stabilized.

For sensing characterization the sensors were placed in a quartz tube inside a horizontal tube furnace (Lindberg Blue M: TF55035COMA1, USA). The sensor was connected to a data acquisition system using Au wire (0.2 mm, Alfa Aesar, Ward Hill, MA, USA). Au paste (Heraeus, Hanau, Germany) was applied to the sensor/wire junction and cured at 650 °C for 1 h in Ar and in ambient environment to attach the lead wire. Before starting sensing experiments, the quartz tube was purged with Ar gas for 10 min to remove possible contaminations. The furnace was heated to 450–650 °C at a heating rate of 30 °C/min in N_2_ environment (purity: 99.999% with <5 ppm O_2_). The target gases (C_2_H_5_OH, CH_4_, H_2_) were mixed with N_2_ at different concentrations ranging from 20 to 1000 ppm by using digital mass flow controller (C100L-CM-NR-2-0V1-SV1-PV2-V1, Sierra, Monterey, CA, USA). The total flow rate of gases was maintained at 500 sccm throughout the experiments. The sensitivity response of the sensor is defined as *R_a_*/*R_g_*, where *R_a_* and *R_g_* is the resistance of the sensor in air and in the target gas, respectively. The response time (*T_res_*) and recovery time (*T_rec_*) of the sensor are defined as the time to reach 90% of the total resistance change in case of gas exposure and removal, respectively. The optimum operating temperature, sensitivity, response and recovery times were also determined for different gases.

## 3. Results and Discussion

**Figure 1.**
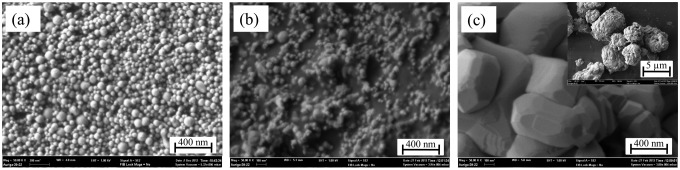
TiO_2_ particles (**a**) in as-received condition; (**b**) heat treated at 750 °C for 3 h and (**c**) heat treated at 1000 °C for 1 h (inset showing low magnification image of the sample).

Figure 1 shows the field emission scanning electron microscope (FESEM) images of TiO_2_ nanoparticles in as-received and heat treated conditions. The global view of the as-received particles indicates that the particles had spherical shape without any agglomeration (Figure 1a). The average size of the as-received particles was about 50 nm under FESEM observation. After heat treatment at 750 °C, the particles size and shape are almost similar to that of the as-received particles (Figure 1b). However, the micrograph suggests that sintering has started to occur during the heat treatment. All TiO_2_ particles sintered together after heat treatment at 1000 °C (Figure 1c). The average size of the sintered particles was 3–5 μm (inset in Figure 1c).

**Figure 2.**
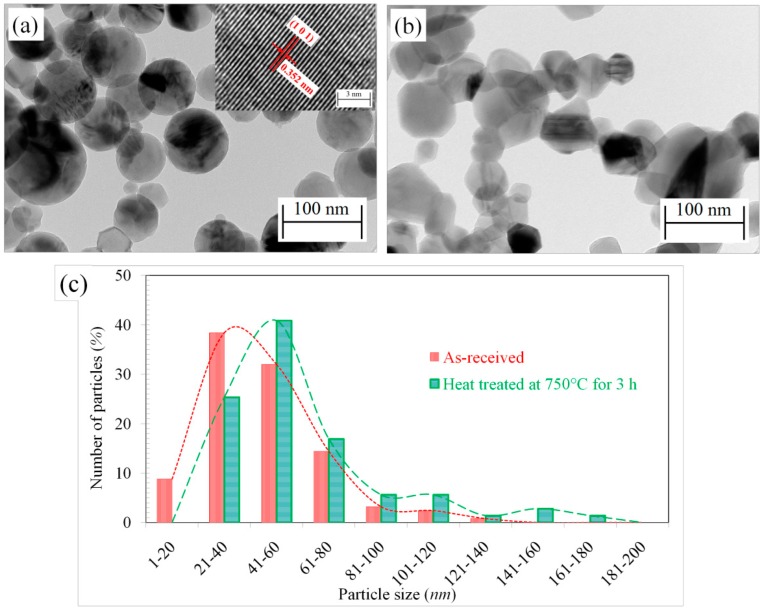
TEM images of the TiO_2_ particles in (**a**) as-received condition; (**b**) heat treated at 750 °C for 3 h and (**c**) size distribution of the particles in as-received and heat treated condition.

Figure 2 shows transmission electron microscope (TEM) images of the as-received particles and those heat treated at 750 °C. The average particle size was calculated from the TEM images. More than 100 particles were used to calculate the average particle size. From the TEM images the average size of the as-received particles are calculated to be 45.3 ± 21.6 nm (Figure 2a). The high resolution TEM analysis shows that the as-received particles are completely crystalline with a mixture of anatase and rutile. One such anatase crystal is shown in the inset of Figure 2a. On the other hand, after heat treatment at 750 °C the particles became faceted, with an average size of 60.1 ± 30.7 nm (Figure 2b). The faceted structure is due to the increased portion of rutile in the particles after heat treatment. The size distribution of the particles is shown in Figure 2c. Particles heat treated at 1000 °C were not transparent to electron beam and were not observable under TEM due to increased size (3–5 μm).

**Figure 3.**
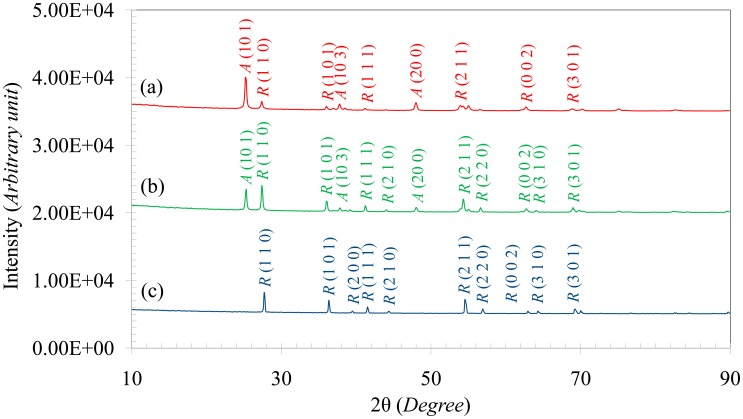
XRD pattern of the TiO_2_ particles (**a**) in as-received condition; (**b**) Heat treated at 750 °C for 3 h and (**c**) heat treated at 1000 °C for 1 h in Ar atmosphere. Here, “*A*” and “*R*” represent anatase and rutile, respectively.

Figure 3 shows the XRD patterns of the TiO_2_ particles in as-received and heat treated conditions. The as-received particles contain both anatase and rutile peaks of TiO_2_ (Figure 3a). After heat treatment at 750 °C the rutile fraction increased (Figure 3b) and heat treatment at 1000 °C completely converted the particles to rutile, as seen in Figure 3c. The anatase and rutile content of the particles were calculated from the XRD patterns by using Spurr and Myers's equation [32] and the results are tabulated in Table 1. The as-received particles contained 76.3% anatase which was reduced to 37% after heat treating at 750 °C for 3 h. After heat treating at 1000 °C, the particles were completely converted to rutile.

The sensors are intended to operate at high temperatures, so it is necessary that the phase content and microstructure of the particles do not change during the test. As it can be seen from the images in Figures 1 and 2 in combination with the XRD results in Figure 3, the particle morphology and rutile content of the as-received particles changed after heat treatment at 750 °C. For this reason, the as-received TiO_2_ particles were excluded from the sensing test. Moreover, after heat treatment at 1000 °C, the particles converted to rutile, with sizes ranging from 500 nm to 1 μm (Figures 1 and 3). The sintered morphology of the particles at 1000 °C greatly reduced the sensing surface. For this reason, these particles were also excluded for sensing tests. Since, the maximum operating temperature of the sensor was 650 °C; the particles heat treated at 750 °C were chosen for sensing tests to ensure morphological and phase stability during sensing.

**Figure 4.**
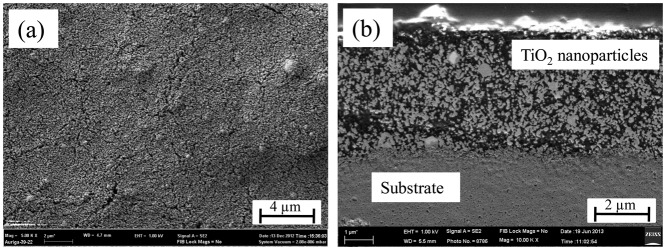
FESEM images of TiO_2_ nanoparticles coated on Au interdigitated alumina substrate followed by heat treatment (**a**) in top view and (**b**) cross sectional view.

For the preparation of the sensor, composite ink were prepared by loading 10 wt% of TiO_2_ nanoparticles in an ink. About 3 μL of composite ink was dropped on to the Au interdigitated alumina substrate and then heat treated at 750 °C for 1 h in Ar and ambient environment to stabilize the particles and create some bonds between the substrate and particles. Investigation under FESEM shows that a more or less homogeneous film was created on top of the substrate (Figure 4a). The cross sectional view of the sensor shows that the film is porous with a thickness of 4.5 μm (Figure 4b). It has been reported that a porous film is beneficial for gas sensing because the accessibility of the target gas to the active surface layer is increased [33].

**Figure 5.**
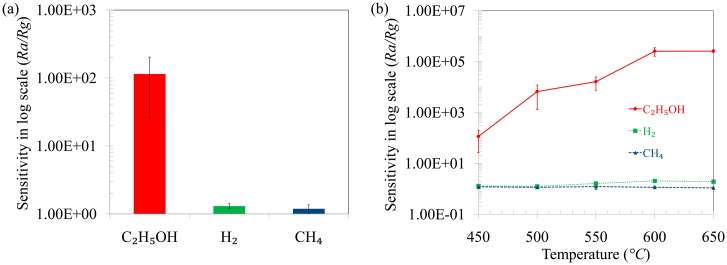
Response towards C_2_H_5_OH, H_2_ and CH_4_ gas for 1000 ppm concentration (**a**) at 450 °C and (**b**) among ethanol, hydrogen and methane gas.

To determine the optimum operating conditions, the sensing temperature was varied from 450–650 °C at 1000 ppm target gas concentration. Figure 5a shows the sensitivity of the sensor for C_2_H_5_OH, H_2_ and CH_4_ gases at 450 °C at 1000 ppm concentration. The sensitivity of C_2_H_5_OH is much higher compared to that of H_2_ and CH_4_ at 450 °C. The sensitivity of the sensor for C_2_H_5_OH is ∼100 whereas it is only ∼1.3 and ∼1.2 for H_2_ and CH_4_, respectively, at 450 °C, so the sensor is capable of detecting C_2_H_5_OH selectively in the presence of H_2_ and CH_4_. The sensitivity of the sensor at different temperatures was also investigated and the results are shown in Figure 5b. The sensors show a much higher sensitivity towards C_2_H_5_OH at all temperatures. Moreover, with increasing temperature, the sensitivity towards C_2_H_5_OH increases until 600 °C and after that it remains more or less constant. On the other hand, the sensitivity towards H_2_ and CH_4_ is very negligible compared to that of C_2_H_5_OH. For example, the sensitivity at 600 °C is ∼2.5 × 10^5^, 2.1 and 1.5 for C_2_H_5_OH, H_2_ and CH_4_, respectively. So, from the above observations it can be stated that the sensor containing TiO_2_ nanoparticles selectively detects C_2_H_5_OH at all temperatures and the optimum temperature is determined to be 600 °C.

**Figure 6.**
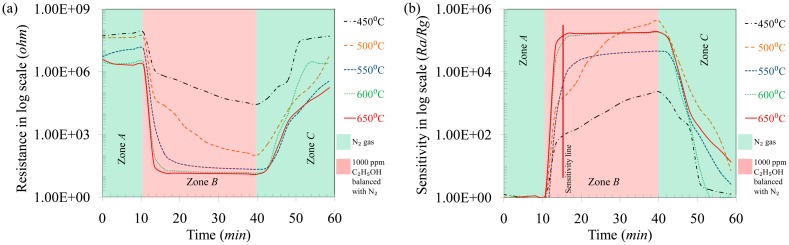
Response of TiO_2_ nanoparticles towards 1000 ppm C_2_H_5_OH at different temperatures: (**a**) resistance curves and (**b**) sensitivity curves.

The response curves of the sensor towards C_2_H_5_OH gas at different temperatures (450–650 °C) are shown in Figure 6, where three distinct zones can be seen. Zone *A* is the region where pure N_2_ gas was flown at a rate of 500 sccm into the sensing chamber. It is clearly seen that the initial resistance of the sensor decreased with increase in the temperature. In zone *B*, 1000 ppm C_2_H_5_OH gas was introduced into the sensing chamber at 500 sccm flow rate. At low temperatures (450–500 °C), the resistance of the sensor was not stable in zone *B* and had a decreasing trend. On the other hand at higher temperatures (550–650 °C), the resistance of the sensor reached a minimum and stabilized to a more or less constant value. Finally, in zone *C*, again pure N_2_ gas was flown into the sensing chamber resulting in the recovery of the initial resistance. The sensitivity (*R_a_*/*R_g_*) of the sensor towards 1000 ppm C_2_H_5_OH gas was calculated from the response curves and is presented in Figure 6b. At low temperatures (450–500 °C) the sensitivity of the sensors towards C_2_H_5_OH was not constant and had an increasing trend as it can be seen in zone *B* in Figure 6b. On the other hand, the sensitivity reached a more or less constant value at high temperatures (550–650 °C) after the introduction of C_2_H_5_OH gas to the sensing chamber. To avoid the ambiguities in sensitivity values, a sensitivity line was drawn after 5 min of C_2_H_5_OH sensing (Figure 6b) and these values are reported in Figure 5b. From Figures 5 and 6, it is seen that the best response for C_2_H_5_OH was obtained at 600 °C and this temperature was chosen as the optimum temperature for the TiO_2_ sensor.

**Figure 7.**
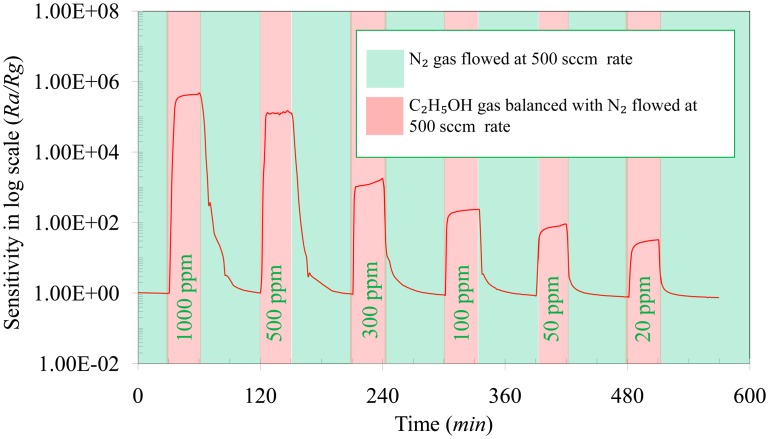
Sensitivity of the sensor towards C_2_H_5_OH at different concentrations (20 ppm–1000 ppm) at 600 °C.

To demonstrate the effect of concentration, C_2_H_5_OH was varied from 1000 ppm to 20 ppm in a N_2_ environment at the optimum operating temperature of 600 °C (Figure 7). It is seen that with decreasing C_2_H_5_OH concentration the sensitivity of the sensor decreases. Within the limit of the experimental facilities it is seen that the sensor is able to detect C_2_H_5_OH levelsas low as 20 ppm in a N_2_ atmosphere with a sensitivity response of 31. It is also seen that the response time (*T_res_*) and recovery time (*T_rec_*) of the sensor decreases with decreasing C_2_H_5_OH concentration. The response time was reduced from 6 min to 3 min with a decrease in the C_2_H_5_OH concentration from 1000 ppm to 20 ppm. Similarly, the recovery time of the sensor decreased from 30 min to 15 min by reducing the C_2_H_5_OH concentration from 1000 ppm to 20 ppm. The sluggish recovery of the sensor, typical of the oxide-based sensors, is possibly due to slow desorption rate of C_2_H_5_OH from the TiO_2_ surface [34].

**Figure 8.**
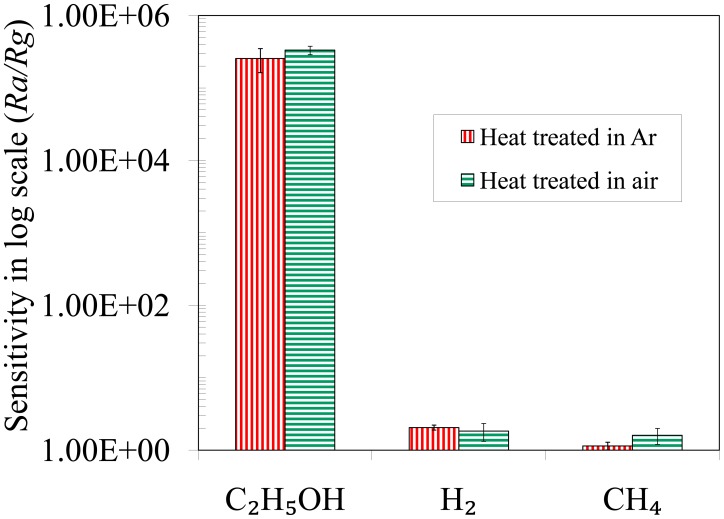
Response of the sensors towards 1000 ppm C_2_H_5_OH, H_2_ and CH_4_ gases after heat treating in Ar and air environment at 750 °C for 3 h.

From the above observation, an ultrahigh response is seen for C_2_H_5_OH detection by using heat treated TiO_2_ nanoparticles in Ar environment at 750 °C. It was assumed that the nanoparticles were partially reduced during the heat treatment in Ar. Partial reduction of the particles increases the oxygen vacancies which in turn results in ultrahigh responses in the sensor. For this reason another set of experiments were conducted by fabricating sensors after heat treating the TiO_2_ nanoparticles in air at 750 °C for 3 h. Figure 8 compares the response of the sensors towards 1000 ppm of C_2_H_5_OH, H_2_ and CH_4_ gases at the optimum operating temperature (600 °C) after heat treating the sensors at 750 °C for 3 h in Ar and air. No obvious change in response is observed from this figure. The responses toward C_2_H_5_OH, H_2_ and CH_4_ gases remain more or less constant in both types of sensors at the optimum temperature, so partial reduction of the nanoparticles during heat treatment in Ar environment is not responsible for the ultrahigh response of the sensor. Other factors such as the thickness of the electron depleted layer, catalytic activity and porosity in the film might be responsible for the ultrahigh response of these sensors.

The mechanism of C_2_H_5_OH sensing by TiO_2_ nanoparticles may be attributed and explained with the help of the surface depletion model [35]. During the exposure of TiO_2_ to ambient environment, oxygen molecules are adsorbed on the surface as O^−^, O_2_^−^ or even O^2−^ ions by capturing an electron from the conduction band. As a result, an electron depleted layer is formed at the surface of TiO_2_. The thickness of electron depleted layer of TiO_2_ is approximately 10–25 nm [16], which is comparable with the radius of the TiO_2_ nanoparticles used in this experiments (average radius of the nanoparticles is ∼30 nm), so it is expected that the major portion of the radius of a nanoparticle is electron depleted. Though a thick film of TiO_2_ was formed during the preparation of the sensor (4.5 μm), presence of high porosity in the film (Figure 4) effectively creates a path for oxygen molecules to reach deep inside the film for the surface reaction. As a result an electron depleted layer is created in each particle which could be a reason for the high resistance of the sensor in a nitrogen environment (on the order of 10^6^ to 10^7^ ohm). Upon exposing the sensor to a reducing environment, the target gases inject electrons into the TiO_2_ resulting in a decrease of the sensor resistance. However, the electron donating capacity of different types of molecules is not the same, and for alkyl groups this capacity increases with the increase of branches. In this regard, C_2_H_5_OH has higher electron donating capacity than CH_4_. This could be a reason for the higher sensitivity towards C_2_H_5_OH compared to CH_4_ and H_2_.

A relevant literature survey on the catalytic action of TiO_2_ nanoparticles reveals that the adsorption of C_2_H_5_OH on the surface of TiO_2_ is largely dissociative and yields ethoxide and surface hydroxyl groups [11]. The C_2_H_5_OH molecules adsorb on the surface of TiO_2_ nanoparticles and desorb as acetaldehyde (CH_3_CHO) with other minor products such as acetone (CH_3_-C(O)-CH_3_) and ethyl acetate (CH_3_CH_2_COOCH_3_). Reactions involved in the evolution of these products are dehydrogenation and coupling. It may be noted that a Au interdigitated alumina substrate was used for making the sensors and it has been reported that the presence of Au is beneficial to improve the catalytic activity of TiO_2_ [12]. In general, the O-H bond in C_2_H_5_OH dissociates and yields ethoxide which donates electron to the TiO_2_ surface. In the present experiment, it is believed that the adsorption kinetics of C_2_H_5_OH are slower at temperatures up to 500 °C compared to higher temperatures (550–650 °C). As a result, the surface slowly accumulates the adsorbed C_2_H_5_OH which results in a prolonged decreasing trend in resistance during the exposure of the target gas. It can be also seen that the resistance of the sensor did not reach to a stable minimum in zone *B* at temperatures of 450–500 °C (Figure 6a) due to slow reaction kinetics. Prolonged exposure to C_2_H_5_OH might result in a stable minimum value of resistance at these temperatures. But in that case, the response time will be significantly high and for this reason these temperatures are not feasible for sensing. On the other hand, at high temperatures (550–650 °C) the reaction kinetics are fast enough for surface saturation by the adsorbed species which leads the resistance of the sensors to reach a minimum value within a reasonable time. For this reason a fast response is observed in the sensors at the temperatures above 550 °C, so it can be concluded that high temperatures (550–650 °C) are beneficial to obtain a stable response from the sensors. The variant slopes in the recovery response shown in the zone *C* of Figure 6a could be due to desorption of different gases at different rates, which changes the resistance of the sensors to different degrees.

So far, different types of metal oxides with different morphologies have been used for detecting C_2_H_5_OH gas and the results are tabulated in Table 2 [36–49]. In comparison with other metal oxides, TiO_2_ exhibits higher response for C_2_H_5_OH due to its higher catalytic reaction and enhanced charge transfer compared to ZnO, SnO_2_, In_2_O_3_ and CuO. Increased response towards C_2_H_5_OH is also observed in ZnO and SnO_2_ nanostructures compared to other reducing gases [10]. Comparing with other TiO_2_ morphologies such as nanowire arrays [50], nanobelts [16] and spherical colloids [30], the spherical nanoparticles of TiO_2_ shows remarkable high sensitivity towards C_2_H_5_OH. This could be due to smaller particle size (60.1 ± 30.7 nm) which is comparable with the electron depletion thickness (10–25 nm) with high surface-to-volume ratio. Moreover, a porous film composed of TiO_2_ nanoparticles makes it easier for oxygen from the ambient to diffuse into the film to create an electron depleted layer by pulling electrons from the surface of the nanoparticles. As a result, all nanoparticles as well as the whole film become electron depleted under ambient conditions. Similarly, any target gas to be detected requires diffusion into the bulk to develop the desired chemical reaction(s). The change in the electrical resistance of the sensing material happens close to the reaction sites. It is obvious that a higher number of reaction sites will provide greater changes in the electrical resistance of sensor device [33]. In this regard a porous film is beneficial since it can provide a passage for the target gas moleculea to diffuse deep inside the sensing film. As it can be seen from Figure 4, a uniform porous film of TiO_2_ was formed in this study and this combined with almost electron depleted particles, higher catalytic activity and enhanced charge transfer are possible reasons for the observed better sensitivity towards ethanol.

## 4. Conclusions

In this research, the sensitivity of TiO_2_ nanoparticles towards C_2_H_5_OH, H_2_ and CH_4_ gases was investigated. For this, a film of TiO_2_ having 4.5 μm thickness was prepared on a Au interdigitated alumina substrate. Before sensing the particles were stabilized by heat treatment at 750 °C with 37% anatase and 63% rutile phase. From the experimental observations of this work the following conclusions can be drawn:

1. TiO_2_ nanoparticles selectively detect ethanol (C_2_H_5_OH) in the presence of hydrogen (H_2_) and methane (CH_4_).
2. The optimum operating temperature of TiO_2_ nanoparticles is 600 °C for C_2_H_5_OH detection.
3. The sensitivity of the sensor towards C_2_H_5_OH, H_2_ and CH_4_ is about ∼2.5 × 10^5^, 2.1 and 1.5, respectively at the optimum operating temperature of 600 °C.
4. The response time (*T_res_*) and recovery time (*T_rec_*) of the senor varies with C_2_H_5_OH concentration. In general, *T_res_* and *T_rec_* decrease with decreasing C_2_H_5_OH concentration. For 20 ppm C_2_H_5_OH detection at the optimum operating temperature, the *T_res_* and *T_rec_* are 3 min and 15 min, respectively.

## Figures and Tables

**Table 1.** Characterization of TiO_2_ particles in as-received and heat treated conditions.

**Treatment of the Particles**	**Particle Size**	**Crystal Structure**	**Phase Content**	

			**Anatase Content (%)**	**Rutile Content (%)**
TiO_2_ as-received	45.3 ± 21.6 nm	Anatase and rutile	76.3	23.7
TiO_2_ heat treated at 750 °C for 3 h	60.1 ± 30.7 nm	Anatase and rutile	37	63
TiO_2_ heat treated at 1000 °C for 1 h	3–5 μm	Rutile	0	100

**Table 2.** Comparison of sensitivity towards C_2_H_5_OH gas by using different metal oxides.

**Material**	**Crystal Structure**	**Morphology**	**Size**		**Sensitivity**	**Test Concentration (ppm)**	**Operating Temperature (°C)**	**Response Tine (sec)**	**Recovery Time (sec)**	**Reference**

			**Diameter**	**Length**						
ZnO	Wurtzite	Nanoparticles	39, 42 nm	-	25 ^a^	1000	370	∼50	∼450	37
	-	Nanowire	25 ± 5 nm	-	32 ^a^	100	300	-	-	38
	Wurtzite	Nanowire	80 nm	1 μm	43 ^b^	100	300	-	-	39
	-	Nanorod (flowerlike)	150 nm	Few micron	14.6 ^a^	100	300	-	-	40
	Wurtzite	Nanorod (bushlike)	15 nm	1 μm	29.7 ^a^	100	300	-	-	41
	Wurtzite	Nanorod (vertically aligned)	50 nm	500 nm	100 ^a^	100	300	-	-	42
SnO_2_	Tetragonal	Nanoparticles	3.3 ± 6 nm	-	∼175 ^a^	500	220	18	44	43
	Tetragonal	Nanowhisker	50–200 nm	Tens of micrometers	23 ^a^	50	300	-	600	44
	Tetragonal	Nanorod (flowerlike)	5–20 nm	100-200 nm	45.1 ^a^	100	200	-	-	45
	Tetragonal	Nanorods (flowerlike loaded with La_2_O_3_)	5–20 nm	100–200 nm	213 ^a^	100	200	-	-	45
	Tetragonal	Nanofiber (Pd doped)	200–300 nm	Tens of micrometers	1,020.6 ^a^	100	330	<10	503	46
In_2_O_3_	Cubic	Nanofiber	60 nm	-	379 ^a^	15,000	300	1	5	47
	-	Nanowire	60–160 nm	0.5 to a few micrometer	25.3 ^a^	1000	370	10	20	48
	Hexagonal	Nanorod	20–50 nm	> 100 nm	11.5 ^a^	50	330	6	11	49
CuO	Monoclinic	CuO Nanoribbon	2–8 nm	30–100 nm	∼3.5^c^	1000	200	3-6	4–9	50
	Monoclinic	CuO Nanoribbon (Au loaded)	2–8 nm	30–100 nm	∼3.5^c^	1000	200	-	-	50
	Monoclinic	CuO Nanoribbon (Pt loaded)	2–8 nm	30–100 nm	∼6^c^	1000	200	-	-	50
TiO_2_	Anatase	Nanowire array	90–180 nm (Width)	1400 μm	50 ^a^	20,000	550	-	-	36
	Anatase	Nanotubes	50 nm	1 μm	∼15 ^a^	47	500	-	-	31
	Anatase	Nanobelt	50 nm (thickness)	100–150 nm (width)	46.153 ^a^	500	200	1-2	1–2	16
	Anatase	Spherical colloids	250 nm	-	15.1 ^a^	10,000	350	115	340	30
	Anatase	Spherical colloids (Ag loaded)	250 nm	-	41.7 ^a^	10,000	350	1	35	30
	37% Anatase and 67% Rutile	Spherical nanoparticles	60.1 ± 30.7 nm	-	2.5 × 10^5^ ^a^	1000	600	180 (20 ppm)	360 (20 ppm)	Present work

Note:

a *S* = *R_a_*/*R_g_*,

b *S*=*[(R_a_-R_g_)*/*R_a_]* × *100*% and

c *S* = *R_g_* /*R_a_*.
